# A tumor-associated autoantibody panel for the detection of non-small cell lung cancer

**DOI:** 10.3389/fonc.2022.1056572

**Published:** 2022-12-02

**Authors:** Ruijun Cai, Feng Zhao, Haiying Zhou, Zengsong Wang, Dang Lin, Lu Huang, Wenling Xie, Jiawen Chen, Lamei Zhou, Ni Zhang, Chaoyuan Huang

**Affiliations:** ^1^ Department of Thoracic Surgery, Nanfang Hospital, Southern Medical University, Guangzhou, China; ^2^ Research and Development Department, Guangzhou BioBlue Technology Co. Ltd, Guangzhou, China; ^3^ Department of Orthopaedics, AIR Force Hospital of Southern Theater Command of People's Liberation Army of China (PLA), Guangzhou, China; ^4^ School of Pharmacutical Sciences, Wuhan University, Wuhan, China; ^5^ Department of Thoracic Surgery, Tongji Hospital, Tongji Medical College, Huazhong University of Science and Technology, Wuhan, China

**Keywords:** SEREX, TAAS, liquid chip, non-small cell lung cancer, early diagnosis

## Abstract

Lung cancer is the second most frequent malignancy and the leading cause of cancer-associated death worldwide. Compared with patients diagnosed at advanced disease stages, early detection of lung cancer significantly improved the 5-year survival rate from 3.3% to 48.8%, which highlights the importance of early detection. Although multiple technologies have been applied to the screening and early diagnosis of lung cancer so far, some limitations still exist so they could not fully suit the needs for clinical application. Evidence show that autoantibodies targeting tumor-associated antigens(TAAs) could be found in the sera of early-stage patients, and they are of great value in diagnosis. Methods, we identified and screened TAAs in early-stage non-small cell lung cancer(NSCLC) samples using the serological analysis of recombinant cDNA expression libraries(SEREX). We measured the levels of the 36 autoantibodies targeting TAAs obtained by preliminary screening *via* liquid chip technique in the training set(332 serum samples from early-stage NSCLC patients, 167 samples from patients with benign lung lesions, and 208 samples from patients with no obvious abnormalities in lungs), and established a binary logistic regression model based on the levels of 8 autoantibodies to distinguish NSCLC samples. Results, We validated the diagnostic efficacy of this model in an independent test set(163 serum samples from early-stage NSCLC patients, and 183 samples from patients with benign lung lesions), the model performed well in distinguishing NSCLC samples with an AUC of 0.8194. After joining the levels of 4 serum tumor markers into its independent variables, the final model reached an AUC of 0.8568, this was better than just using the 8 autoantibodies (AUC:0.8194) or the 4 serum tumor markers alone(AUC: 0.6948). In conclusion, we screened and identified a set of autoantibodies in the sera of early-stage NSCLC patients through SEREX and liquid chip technique. Based on the levels of 8 autoantibodies, we established a binary logistic regression model that could diagnose early-stage NSCLC with high sensitivity and specificity, and the 4 conventional serum tumor markers were also suggested to be effective supplements for the 8 autoantibodies in the early diagnosis of NSCLC.

## 1 Introduction

Lung cancer is the most common and deadly cancer in both China and the world (1, 2). It is high mortality will persist for a long time to come unless substantial progress is made in the areas of early detection, treatment, and prevention. Currently, the main screening and diagnostic methods for lung cancer include CT, bronchoscopy, lung needle biopsy, and serological test. Among them, CT has a high false positive rate,. It was found that up to 30% of resected nodules found by lung cancer CT screening were diagnosed as benign by histopathology (3); especially for small pulmonary nodules (less than 3 mm), and the false rate could be further increased in the case of diagnosis by CT (4–6). Currently, traditional tumor markers are mainly used for treatment efficacy and prognosis monitoring, its sensitivity and specificity in adjuvant diagnosis cannot meet the clinical diagnostic requirements (7). Furthermore, lung biopsy and bronchoscopy are invasive, which have poor patient compliance and low positive diagnostic rates. Serology ctDNA test has been used extensively in lung cancer recurrence monitoring and driver gene detection (8, 9), and has also been explored for early diagnosis of lung cancer. GRAIL, a US-based company in the field of early cancer screening, has conducted the CCGA (Circulating Cell-free Genome Atlas) project, and one of its subprojects has been presented at the ASCO Congress. In this project, researchers evaluated the effects of targeted sequencing, whole genome sequencing, and whole genome methylation sequencing of cfNA (cell-free nucleic acid) on the early diagnosis of lung cancer. It showed that, while these three technologies achieved 87–89% sensitivity in advanced lung cancer (stages IIIB–IV), they had only 38–51% sensitivity in early-stage lung cancer (stages I–IIIA), and that the detection process was complex and expensive. Thus, the assay of cfNA still does not meet the demand for early diagnosis of lung cancer. Therefore, there is an urgent clinical need for non-invasive early lung cancer detection methods with both high specificity and sensitivity.

In the early stage of tumor development, tumor-associated antigens (TAAs) are produced due to gene mutation, abnormal expression, or abnormal modification of proteins. In addition, the death and lysis of tumor cells also release TAAs, which can stimulate the body to produce antibodies, called tumor-associated autoantibodies (10). The tumor associated autoantibodies produced by the body’s immune system are stable, have a long half-life, and can exist continuously and stably in the serum. When stored for a long time at -20°C, their physicochemical properties do not change obviously, which helps to ensure the stability of antibody assay results. Studies have demonstrated that tumor-associated autoantibodies, such as those targeting p53, SOX-2, and CAGE-1, can be detected in the sera of patients at the early stage of tumorigenesis. Autoantibodies have high titers in the serum because, compared to tumor antigens, their abundance amplified many folds with the effect of the immune system, resulting in easy detection (10). Based on the above characteristics, tumor-associated autoantibodies have great potential for lung cancer diagnosis. In recent years, attention has been paid to tumor-associated autoantibodies in clinical diagnosis. The results of some preliminary small sample studies suggest that autoantibodies for lung cancer have a good diagnostic value in early diagnosis. A study by Titulaer, M. J. et al. showed that serum levels of autoantibodies such as anti-SOX are elevated in patients with non-small cell lung cancer (NSCLC), and these autoantibodies can be used as serological markers to more effectively distinguish patients with NSCLC from healthy individuals (11). In addition, due to heterogeneity among tumors (12, 13), different individuals may have different tumor-associated autoantibody profiles, even for the same subtype of the same kind of cancer, so the diagnostic efficacy of an individual tumor antibody is usually limited. At present, the mainstream testing mode for tumor autoantibodies is the combined detection of multiple autoantibodies. Recently, early diagnosis of lung cancer based on autoantibody profiles has gradually shown its value in clinical application. Wang, J. et al., using a combination consisting of 5 autoantibodies, distinguished lung cancer from benign lung nodules in a relatively effective manner (14). Oncimmune, a UK-based company, has launched EarlyCDT Lung test, a lung cancer early detection product based on the detection of 7 autoantibodies (CAGE, GBU4-5, HuD, MAGE A4, NY-ESO-1, p53, and SOX2) presenting in the sera of lung cancer patients. Four large-scale prospective clinical studies involving 15,000 subjects have shown that EarlyCDT has a sensitivity of 57% and a specificity of 88–90% in detecting early stage NSCLC, and that it is a validated model as well as an effective complement to CT scans for the early detection of lung cancer. Although showing promising results for the early diagnosis of lung cancer *via* autoantibodies, the assays in the above studies were performed using ELISA that could only detect small numbers of markers, usually no more than 7, due to low throughput and narrow linearity range. It prevents any further improvement in the sensitivity and specificity of the above tests, limiting their clinical application.

Liquid chip technique, represented by Luminex, is more and more commonly used for immune or nucleic acid tests in recent years and is widely used for the assay of cytokines, antibodies, or nucleic acid mutations. Compared with conventional ELISA, the liquid chip technique is more reproducible, has higher sensitivity and wider linearity range, and more importantly, allows the simultaneous co-detection of up to 500 indicators (FlexMap3D), meeting the need for multi-indicator, high-throughput assays (15). In this study, Luminex liquid chip technology was used to detect lung cancer autoantibodies in serum samples for screening and detection of lung cancer-associated antigens.

SEREX (serological analysis of recombinant cDNA expression libraries) is a new method for serological tumor antigen detection developed by German serologist Sahin et al. in 1995 (16). In this method, a patient’s serum is used to identify tumor antigens at the molecular level by screening and searching for tumor antigen genes from the cDNA library of the patient’s tumor cells based on the reaction of the antigen with the autologous serum. The tumor antigens screened by SEREX not only include tumor cell surface antigens but also cover most of the proteins encoded by tumor-associated genes. What’s more, using diluted serum from patient for screening can limit the immunoassay to only those antigens that induce strong immune responses, so it is a powerful means of tumor antigen screening and has been applied to a variety of tumors such as lung, liver, and gastric cancers (17–19), and thousands of TAAs have been identified so far.

In this study, lung cancer-associated antigens were screened, identified, and isolated from a cDNA library of lung cancer using SEREX technology, and high-throughput screening *via* liquid chip technique was performed for up to 36 lung cancer-associated antigens obtained by initially screening in the training set samples. Finally, autoantibodies targeting 8 lung cancer-associated antigens were selected by statistical analysis and used as a lung cancer autoantibody profile. Based on the autoantibody profile, a prediction model was constructed and validated in the test set samples, showing its efficacy in differentiating between sera from patients with early-stage lung cancer and sera from patients with benign space-occupying lung lesions. This study identified a combination of serological markers with high potential for clinical translation in the early diagnosis of lung cancer, and also identified some lung cancer TAAs, which provide potential targets for targeted therapy in lung cancer.

## 2. Materials and methods

### 2.1 Serum samples and main reagents

Tissues used for mRNA extraction in this study were derived from surgically resected specimens from 10 stages I lung cancer patients (4 with adenocarcinoma, 4 with squamous carcinoma, and 2 with large-cell carcinoma), and they were frozen in liquid nitrogen immediately once obtained. Information on the 30 early-stage allogeneic NSCLC samples used for cDNA library screening is described in **Table 1**. The sera used for the screening of marker combinations and the establishment of diagnostic models are referred to as the training set samples, which contained 185 sera from treatment-naive patients with initial diagnosis of early-stage lung cancer, 74 sera from patients with benign space-occupying lung lesions who were excluded from lung cancer, and 89 sera from subjects with no obvious lung abnormalities in the physical examination. The information on the training set samples is described in **Table 2**. The sera used to validate the diagnostic efficacy of the screened marker combinations are referred to as the test set samples, which contained 163 sera from treatment-naive patients with initial diagnosis of early-stage lung cancer and 183 sera from patients with benign space-occupying lung lesions who were excluded from lung cancer. Information on the test set lung cancer samples is described in **Table 3**. The abovementioned samples were collected at Nanfang Hospital and the Department of Thoracic Surgery of Wuhan Tongji Hospital, and all sera were collected and stored at -80 °C prior to any processing procedures. All donors signed the informed consent form for sample collection. The main reagents used in this study are described in **Table 4**.

**Table 1:** Information on lung cancer samples used for library screening.

Project name	Subproject	Number/percentage
Lung cancer	Adenocarcinoma	17 (56.7%)
	Squamous carcinoma	9 (30%)
	Adenosquamous cell carcinoma	3 (10%)
	Large-cell carcinoma	1 (3.3%)
Gender	Male	18 (60%)
	Female	12 (40%)

**Table 2:** Information on lung cancer samples in the training set.

Project name	Subproject	Number/percentage
Lung cancer	Adenocarcinoma	195 (58.7%)
	Squamous carcinoma	102 (30.7%)
	Other cancers	35 (10.5%)
Stage	Stage I	126 (38%)
	Stage II	206 (62%)
Gender	Male	148 (44.6%)
	Female	185 (55.4%)

**Table 3:** Information on lung cancer samples in the test set.

Project name	Subproject	Number/percentage
Lung cancer	Adenocarcinoma	89 (54.6%)
	Squamous carcinoma	51 (31.3%)
	Other cancers	23 (14.1%)
Stage	Stage I	67 (41.1%)
	Stage II	96 (58.9%)
Gender	Male	69 (42.3%)
	Female	94 (57.7%)

**Table 4:** Main reagents.

Name	Manufacturer
PolyATtract System 1000 mRNA extraction kit	Promega
THERMOScript™ RT-PCR System	Gibco/BRL
ZAP-cDNA systhesis kit	Stratagene
ZAP-cDNA Gigapack III Gold Cloning kit	Stratagene
α-32p-dATP, α-32p-dCTP 3000 ci/mM	Beijing Yahui Company
CNBr-Sepharose 4B	Amersham
IPTG	Merk
Nitrocellulose membrane	Pall
Goat anti-human IgG-AP	Sigma
NBT/BCIP	Sigma
EcoRI	New England Biotech
XhoI	New England Biotech
DNA small amount extraction kit	Promega
DNA recovery kit	BioDev-Tech. Co., Ltd
EcoRI	NEB
XhoI	NEB
IPTG	Merk
Ni-NTA His-Bind Resins	Novagen
Mouse anti-(His) antibody	Pharmacia
Goat anti-mouse IgG-HRP	Sigma
M-MLV	Invitrogen
Oligo(dT)_15_	Promega
dNTP	Promega
RNasin	Promega
Taq enzyme	Promega
xMAP^®^ Antibody Coupling Kit	Luminex
Magplex^®^ Microspheres	Luminex
R-Phycoerythrin AffiniPure F(ab’)2 Fragment Donkey Anti-Human IgG (H+L)	Jackson ImmunoResearch

### 2.2 Experimental method

#### 2.2.1 Extraction of mRNA from mixed tissues of lung cancer

This was performed following the instructions of mRNA PolyATtract System 1000: 10 tumor tissue specimens were taken at 200 mg each and homogenized, then GTC/β-mercaptoethanol buffer was added, and pre-heated dilution buffer/β-mercaptoethanol at 70°C and Oligo dT probe were added after sufficient cell lysis. Then they were put in a water bath at 70°C for 5 min after being mixed well. After centrifugation at 12,000 rpm for 10 min, the supernatant was collected and incubated with SA-PMPs magnetic beads for 2 min at room temperature. The magnetic beads SA-PMPs were captured *via* the magnetic stand, the supernatant was discarded, then the magnetic beads were washed three times with 0.5 × SSC. After mRNA was eluted using RNase-free deionized water, 0.1-fold volume of 3M NaAc and 1-fold volume of isopropanol were added and precipitated overnight at -20°C. After centrifugation at 12,000 rpm for 20 min, the precipitate was collected, which was then washed with two-fold volume of 70% ethanol and dissolved in RNase-free deionized water. The values of OD260 and OD280 were measured to identify the concentration and purity. 1% agarose gel electrophoresis was performed to analyze the mRNA quality.

#### 2.2.2 Construction of cDNA library for mixed tissues of lung cancer

The 10 extracted mRNAs were mixed in equal proportions and the mixed mRNAs were used as the template for cDNA library construction. The construction was carried out following the instructions of the Stratagene cDNA library construction kit, but the reverse transcriptase and buffer in this kit were replaced by those in the THERMOScriptTM RT-PCR System kit. 1 μL of cDNA was taken and subjected to 1% alkaline agarose gel electrophoresis together with the previously established first-strand synthesis control, and radiographic autoradiography was performed after drying the gel to examine the size and quality of cDNA synthesis. T4 ligase was added to the synthesized cDNA, and the blunt end was connected to an adaptor containing the EcoRI restriction enzyme cutting site, and the adaptor end was phosphorylated using T4 polynucleotide kinase. After being digested with XhoI and precipitated by ethanol, the cDNA was dissolved in 1 × STE buffer. The cDNA fragments were separated chromatographically through a Sepharose CL-2B gel column and collected into separate tubes by the size of the cDNA fragments. All samples were subjected to 5% non-denaturing polyacrylamide gel electrophoresis, and radiographic autoradiography was performed after drying the gel to identify the size of cDNA in each collection tube. The cDNA fragments above 400 bp were pooled and extracted using phenol/chloroform. After precipitation with ethanol, the cDNA precipitates were collected by centrifugation and dissolved in a certain amount of deionized water. The obtained cDNA was quantified using EB plates by comparing with standards of known DNA concentration. The obtained cDNA fragments were ligated to the Uni-ZAP XR vector overnight at 12°C in the presence of T4 ligase in a certain ratio (1:10). 1 μL of ligation product was packaged with ZAP-cDNA Gigapack III Gold Cloning kit following the instructions. The unamplified cDNA phage expression library was created by adding 500 μL of SM buffer after the packaging was completed. 1 μL of the unamplified cDNA library was taken and infected with pre-cultured XL1-Blue-MRF-host bacteria, and then spread on agar plates containing NZY medium. The volume of unamplified library was calculated based on the number of phage plaques grown according to the following formula: library volume = (number of phage plaques × dilution factor of library at the time of infection × total packaging volume)/volume of the library used to infect the host bacteria.

#### 2.2.3 Screening of cDNA library

##### 2.2.3.1 Preparation of *E. coli*/phage lysate solution

Monoclonal XL1-Blue-MRF’ host bacteria were picked from tetracycline plates and cultured in LB medium containing MgSO_4_ and maltose until OD600 was between 0.4 and 1.0. The bacteria were collected after centrifugation at 3000 rpm for 10 min and resuspended in 10 mM MgSO_4_ until the OD600 was about 0.5. 600 μL of the bacterial solution was taken and added with 7 × 10^3^ pfu of empty λ-ZAP vector phages and incubated at 37°C for 15 min, and then 8 mL of NZY/agarose top layer gel was added. After being mixed well, the bacteria were spread on plates with a diameter of 18 cm containing NZY/agar bottom layer gel and incubated overnight at 37°C for amplification. Then 10 mL of coupling buffer (0.1 M NaHCO_3_, pH 8.3) was added to the plate and then eluted by oscillation at 4°C for 16 h. The eluate was collected and the incompletely lysed *E. coli* was ultrasonically broken. Protein quantification was performed by UV spectrophotometry.

##### 2.2.3.2 Absorption serum specimens and validation

The *E. coli*/phage lysate solution were coupled to CNBr-Sepharose 4B column following the instructions, and the serum was diluted 1:9 with 1 × TBS containing 1% BSA and mixed with the column coupled with *E. coli*/phage lysate protein in a volume ratio of 1:1. After gentle oscillation at 4°C overnight, the serum was collected. Following sufficient washing of the gel column with TBS, 5 column volumes of glycine-hydrochloric acid buffer (pH 2.8) was added to wash away unbound antibodies and the column was immediately equilibrated with 1 × TBS. The serum specimen was mixed with the regenerated column material. The above absorption steps were repeated 3 times. The absorbed serum was collected and diluted with 1 × TBS containing 1% BSA at a final dilution of 1:50, preserved by adding 0.02% sodium azide and stored at 4°C for further use. The empty λ-ZAP vector phage was infected with XL1-Blue-MRF’ host bacteria and co-spread on plates with a diameter of 90 mm containing NZY/agar bottom layer gel. It was amplified and cultured at 37°C until the phage plaques were just visible and then the blot transferred to nitrocellulose membranes soaked with 10 mM IPTG to induce expression at 37°C for 8 h. The plate was moved to 4°C and stood for 2 h. After being removed, the nitrocellulose membrane was washed 5 times with TBST buffer and blocked overnight with 1% BSA/TBS at 4°C. The nitrocellulose membrane was then divided into two parts and reacted with the pre-absorbed and post-absorbed sera at room temperature for 1 hour, respectively. The membrane was washed 5 times with TBST and then incubated with alkaline phosphatase-labeled goat anti-human IgM secondary antibody diluted 1:2500 for 1 h at room temperature. After 5 times of washing with TBST, and a last wash performed with TBS, the membrane was added to the prepared NBT/BCIP substrate solution and developed in the dark room. The background reactivity of nitrocellulose membranes before and after serum absorption was compared.

##### 2.2.3.3 First-round screening of cDNA library

Ten autologous mixed sera were diluted 1:50 with a dilution solution containing TBST, 0.02% NaN_3_, and 1% BSA. After the nitrocellulose membrane blot transferred with phage recombinant expression protein reacted with autologous sera at room temperature for 2 h, the nitrocellulose membrane was washed 5 times with TBST, incubated with alkaline phosphatase-labeled goat anti-human IgG secondary antibody diluted 1:2500 for 1 h at room temperature. After 5 times of washing with TBST, and the last wash was performed with TBS, the membrane was then added to the NBT/BCIP substrate solution and developed in the dark room. The second screening was performed on the plaques of positive immunoreactive proteins using goat anti-human IgG antibody to exclude clones that reacted directly with goat anti-human IgG antibody, and the remaining clones were the positive phage clones obtained from the first round of cDNA library screening.

##### 2.2.3.4 Second-round screening of cDNA expression library

The titer of positive phage clones from the first round of screening was titrated, and the positive phage clones were infected with XL1-Blue-MRF’ host bacteria at 800 pfu and co-spread on the plate containing NZY/agar bottom layer gel at 37°C for amplification. When the phage plaques were just visible, the nitrocellulose membrane soaked with 10 mM IPTG were spread on the top layer of gel and expression was induced at 37°C for 4 h. The plates were moved to 4°C and stood for 2 h. After being removed, the nitrocellulose membrane was preserved at 4°C overnight in TBS buffer. Another nitrocellulose membrane soaked with 10 mM IPTG was immediately laid on the top layer of gel, and the above steps were repeated twice. Therefore, three nitrocellulose membranes blot transferred with phage recombinant expression protein were obtained from one plate. All membranes were washed 5 times with TBST buffer and blocked overnight at 4°C in blocking solution. For the three membranes on the same plate, one reacted with 30 sera of early allogeneic lung cancer following the first-round screening method, another reacted with 30 sera from healthy subjects, and the last one reacted directly with goat anti-human IgG. The development of the three membranes was carried out simultaneously in a dark room. Phage plaques that showed negative results after reaction with early lung cancer sera, positive results after reaction with normal sera, and positive results after direct reaction with goat anti-human IgG were recombinant clones with recombinant human immunoglobulin gene sequences, which were therefore removed as false positives. Phage plaques that showed positive results after reaction with early lung cancer sera, negative results after reaction with normal sera, and negative results after reactions with goat anti-human IgG were the true positive clones in the second round of screening of the library. The corresponding positive phage plaques were scooped out, placed in SM buffer, added with 25% chloroform, and stored at 4°C.

#### 2.2.4 Analysis of positive clones

Monoclonal XL1-Blue-MRF’ host bacteria were picked from tetracycline plates and cultured in LB medium containing MgSO_4_ and maltose until OD600 was between 0.4 and 1.0. Bacteria were collected after centrifugation at 3000 rpm for 10 min, and the bacterial precipitate was resuspended with 10 mM MgSO_4_ to an OD600 of approximately 1.0. 200 μL of bacterial solution was taken and added with appropriate amounts of positive monoclonal phages and ExAssist helper phages. After being incubated at 37°C for 15 min, they were added with 3 mL of LB medium and incubated at 37°C with vigorous oscillation for 3 h. The bacteria were inactivated by heating at 70°C. After centrifugation, the supernatant was pipetted out and stored at 4°C. The supernatant contained the pBluescript phages present in the form of single-stranded filamentous phages that had been cleaved. Monoclonal SOLR bacteria were picked from kanamycin plates and cultured in LB medium containing MgSO_4_ and maltose until OD600 was between 0.4 and 1.0. Bacteria were collected after centrifugation at 3000 rpm for 10 min and then resuspended with 10 mM MgSO_4_ to an OD600 of 1.0. 200 μL of bacterial solution was taken and added with a certain amount of cleaved phage supernatant, and incubated at 37°C for 15 min. They were then spread on ampicillin-resistant LB agar plates and incubated upside down at 37°C overnight. The monoclonal bacteria were selected and amplified in LB medium. A portion was used for strain cryopreservation, and another portion was used for small amount plasmid extraction by alkaline lysis. Double digestion was performed with EcoRI and XhoI to preliminarily identify the size of the insert, and the phage clones identified as containing recombinant cDNA fragments were sequenced and then compared in database to determine the gene sequences of positive clones.

#### 2.2.5 Construction and purification of prokaryotic expression vectors for lung cancer antigens

Based on the sequencing and comparison results, the CDS sequences of each gene were obtained from Genbank, and the c-myc and His tags were added on both sides of the gene. The genes were inserted into the PET30A vectors by gene synthesis and transferred into BL21 (DE3) to induce expression after validating the sequences of the recombinant vectors. Positive clones were seeded on agar plates containing kanamycin, and monoclonal colonies were picked and seeded in LB medium for amplification. After induction with IPTG, the bacterial precipitates were harvested, sonication buffer was added, the bacteria were sonicated in an ice bath to break them up, and the inclusion bodies were precipitated by centrifugation. After the inclusion body proteins were weighed, 1 mL of 6M guanidine hydrochloride denaturing solution was added per 100 mg of inclusion body for full denaturation. The supernatant was collected after centrifugation and binded with the treated Ni-NTA His-Bind Resins column material. After the column material was washed with urea-denaturing buffers of pH 8.0 and pH 6.5, the proteins were eluted with urea-denaturing buffers of pH 5.9, pH 5.4, and pH 4.5 sequentially. These eluted proteins were collected in separate tubes and then identified by SDS-PAGE electrophoresis.

#### 2.2.6 Liquid protein chip assay

Biotin-BSA was coupled to the surfaces of Magplex^®^ Microspheres with different numbers following the instructions of xMAP^®^ Antibody Coupling Kit. The lung cancer associated antigens expressed in this study were fused with streptavidin tags and anchored to the surfaces of Magplex^®^ Microspheres with different numbers *via* Biotin-BSA with the help of the high affinity and high specificity of the Biotin-Streptavidin system. In this way, liquid protein chips for detection of autoantibodies associated with lung cancer were prepared. After incubation of the above prepared magnetic beads with serum samples, the lung cancer autoantibodies in the serum samples specifically bound to the corresponding lung cancer associated antigens on the magnetic beads, and the “magnetic bead-lung cancer associated antigen-autoantibody-fluorescent antibody” conjugate was formed by adding R-Phycoerythrin AffiniPure F(ab’)_2_ Fragment Donkey Anti-Human IgG (H+L). The fluorophore of the R-Phycoerythrin AffiniPure F(ab’)_2_ Fragment Donkey Anti-Human IgG (H+L) could be excited by the instrument and receive a fluorescent signal. The intensity of the fluorescent signal was positively correlated with the concentration of lung cancer autoantibodies in the serum samples. Liquid chip magnetic beads for detection of lung cancer autoantibodies were prepared according to the abovementioned method, and the levels of autoantibodies in serum samples were detected.

#### 2.2.7 Analytical method

In this study, the levels of 36 lung cancer-associated autoantibodies in the training set samples were analyzed to initially screen markers with remarkable diagnostic significance for lung cancer using a one-way logistic regression model under 5-fold cross-validation. Further, all possible combinations of markers were listed for the screened markers by means of computerized exhaustive enumeration, and their binary logistic regression models were developed to find the marker combination with the highest diagnostic efficacy based on 5-fold cross-validation.

After the combination of markers with the highest diagnostic efficacy in the training set and the binary logistic regression model built on it were identified, the diagnostic performance of the model was further validated in the test set samples. To further improve the diagnostic performance of the model in this study, the levels of 4 conventional serological tumor markers, CYFRA21-1, CEA, SCCA, and NSE, were additionally measured from the training set samples. After the levels of these 4 markers were incorporated into the model, the benefit of combining lung cancer autoantibodies with these 4 conventional serological tumor markers for the diagnosis of lung cancer was analyzed.

In this study, R 4.1.0 was used for statistical analysis and graphical output of the analysis results.

## 3 Results

### 3.4 Construction of cDNA library for lung cancer tissue

**Figure 1:**
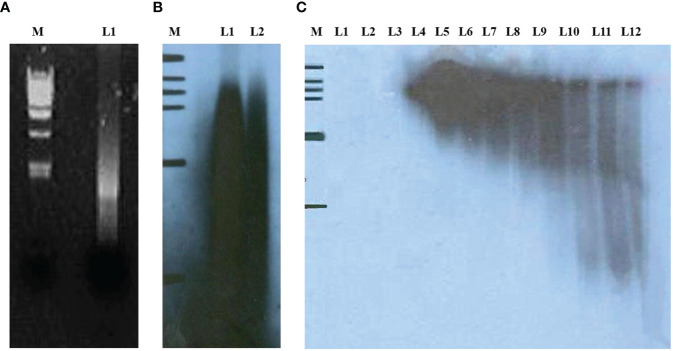
**(A)** L1 indicates mRNA extracted from 10 lung cancer tissues and mixed in equal amounts; **(B)** radiographic autoradiographs of the first and second strands of cDNAs after alkaline agarose gel electrophoresis; **(C)** radiographic autoradiographs of cDNA fragments separated chromatographically and collected by Sepharose CL-2B gel column after non-denaturing polyacrylamide gel electrophoresis.

The cDNA expression library of lung cancer tissues was established by applying SEREX technology. First, the mRNAs extracted from 10 early lung cancer tissues were mixed in equal amounts. **Figure 1A** shows the electropherograms of mRNAs extracted from 10 early lung cancer tissues after being mixed in equal amounts. The above mRNAs were used for reverse-transcription to obtain the first and second strands of cDNAs. **Figure 1B** shows the radiographic autoradiograms of the first and second strands of cDNAs after alkaline agarose gel electrophoresis. The above reverse transcribed cDNA fragments were further separated chromatographically and collected through a Sepharose CL-2B gel column. **Figure 1C** shows the results of radiographic autoradiography of cDNA fragments separated chromatographically and collected using Sepharose CL-2B gel column after non-denaturing polyacrylamide gel electrophoresis. Electrophoresis showed that the fragments in the first 9 tubes (L4–L12) were greater than 400 bp in length, which were then combined and dissolved in 5 μL of deionized water. Its concentration was identified as 75 ng/μL. 100 ng was taken and ligated with ZAP Express vector and then packaged with GigapackIII Gold Cloning kit to obtain an unamplified human lung cancer tissue cDNA expression library with a volume of 3.8 × 10^6^ pfu.

### 3.5 Screening of cDNA library

**Figure 2:**
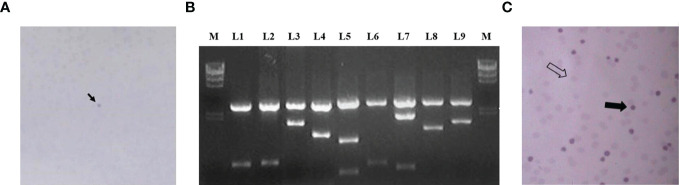
**(A)** The arrow shows the reaction of a representative positive clone on nitrocellulose membrane in the first round of screening; **(B)** L1–L9 are the electrophoresis results of 9 positive clones after double digest; **(C)** shows the results of the second round of screening, the thick arrows indicate positive clones and the empty arrows indicate negative clones.

The obtained cDNA library was transformed into bacteria that were cultured on 10 cm petri dishes until the bacterial plaque was visible. A total of about 3.8 × 10^6^ clones were prepared and 200 petri dishes were used for antigen display, with about 500 bacterial plaques per petri dish, for a total of about 1 × 10^6^ clones. The bacterial plaques were induced with IPTG for antigen expression and the expressed antigens were blot transferred to nitrocellulose membranes, which were then screened using absorbed autologous serum mixes. **Figure 2A** shows the reaction results of a representative positive clone on nitrocellulose membrane. After those clones that reacted directly with goat anti-human IgG antibody were excluded, 78 positive clones were finally obtained. These clones were identified by internal enzymatic digestion, and all of them contained exogenous insert fragments. **Figure 2B** shows the agarose gel electropherograms of 9 positive clones after double digest by EcoRI and XhoI. Homology analysis of protein sequences were conducted by subsequent sequencing, and it was shown that 54 antigen sequences out of 78 positive clones were homologous to 54 human proteins. The 54 clones mentioned above were selected, mixed in equal proportions with the empty vector clones, and spread together on the plates. A second-round screening was performed for 54 positive recombinant clones using 30 allogeneic early-stage NSCLC serum samples and 30 healthy serum samples following the library screening method described above. **Figure 2C** shows the development of a representative positive clone on nitrocellulose membrane in the second-round serum screening. Due to the small sample size in the second-round library screening and the presence of some positive clones with an expected frequency of T < 5, the significance analysis of the serum screening results of the 54 positive clones were uniformly performed using the Yates’ correction for continuity, and the acceptability of the significance level was set to a one-sided 90% CI. Thirty-six clones with a significantly higher true-positive rate than false-positive rate (p < 0.1) were finally obtained in the second-round screening, and the results of these 36 positive clones in the second-round screening are shown in **Table 5**.

**Table 5:** Results of 36 positive clones in the second round of serum screening.

Clone number	True positives^a^	False positives^b^	P value^c^	Clone number	Truepositives	False positives	P value
Clone 1	12	5	0.043	Clone 19	9	3	0.053
Clone 2	4	0	0.060	Clone 20	5	1	0.098
Clone 3	8	3	0.091	Clone 21	4	0	0.060
Clone 4	7	2	0.074	Clone 22	5	0	0.031
Clone 5	4	0	0.060	Clone 23	13	5	0.024
Clone 6	5	1	0.098	Clone 24	8	3	0.091
Clone 7	9	3	0.053	Clone 25	4	0	0.060
Clone 8	9	2	0.022	Clone 26	5	1	0.098
Clone 9	10	3	0.030	Clone 27	11	3	0.016
Clone 10	5	1	0.098	Clone 28	9	3	0.053
Clone 11	8	1	0.015	Clone 29	10	4	0.064
Clone 12	4	0	0.060	Clone 30	6	1	0.054
Clone 13	5	1	0.098	Clone 31	4	0	0.060
Clone 14	12	4	0.021	Clone 32	9	3	0.053
Clone 15	6	1	0.054	Clone 33	6	1	0.054
Clone 16	11	3	0.016	Clone 34	12	5	0.043
Clone 17	8	2	0.042	Clone 35	5	1	0.098
Clone 18	7	2	0.074	Clone 36	8	3	0.091

a: positive samples = 30.

b: negative samples = 30.

c: Yates’ correction for continuity, true positives compared to false positives, 90% CI was accepted.

### 3.6 Recombinant expression and purification of lung cancer antigens

**Figure 3:**
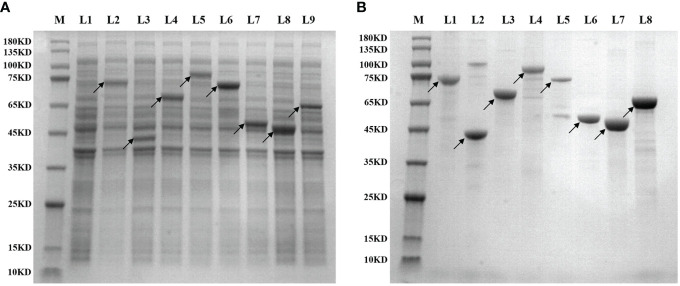
**(A)** shows the expression identification of 8 recombinant lung cancer antigen expressing strains after induction by IPTG, where L1 is the BL21 strain containing the blank plasmid, L2–L9 are the induced expression identification of CCNB1, ESO1, ELAVL3, GAD2, p53, PRDX6, PGP9.5, and SOX2, respectively; **(B)** shows the purification identification of the corresponding 8 recombinant lung cancer antigens.

The 36 clones obtained from the two library screenings represent 36 expression proteins. The CDS sequences of the corresponding mRNAs of these proteins were ligated into the His-tagged PET30a plasmid, respectively, and the streptavidin sequence was then placed at the C-terminal. Then the plasmid was introduced into BL21 (DE3) for induction of expression. **Figure 3A** shows the expression identification of the 8 recombinant lung cancer antigen-expressing strains after induction by IPTG, where L1 is the BL21 strain containing the blank plasmid, and L2–L9 are the electropherograms of the 8 expressing strains after induction of expression, respectively, and the electrophoresis results show that expression was achieved in all the expressing strains after induction (indicated by arrows). Subsequently, 36 recombinant lung cancer antigens were successfully obtained by purification using Ni-affinity chromatography columns. **Figure 3B** shows the purification identification of 8 out of the recombinant lung cancer antigens, which had a purity of more than 80% as analyzed by SDS page electrophoresis. Since the streptavidin tag was designed on the C-terminal of 36 lung cancer antigens, the recombinant lung cancer antigens can be specifically anchored to the surface of MagPlex^®^ Microspheres *via* the biotin-streptavidin affinity system.

### 3.4 Analysis of serum test data

#### 3.4.1 Large sample screening of lung cancer antigens

**Figure 4:**
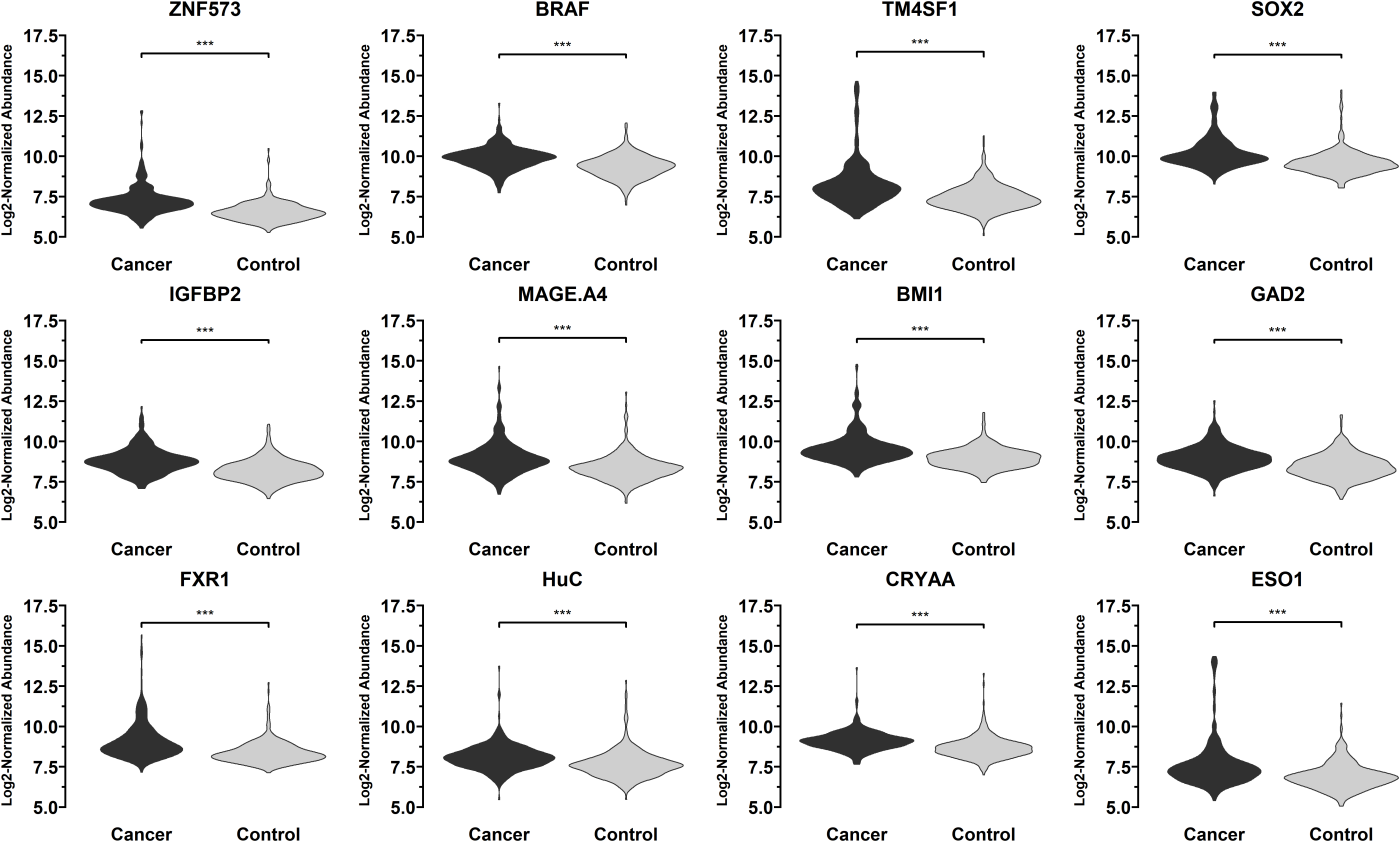
Distribution of the levels of autoantibodies targeting 12 lung cancer antigens (ZNF573, BRAF, TM4SF1, SOX2, IGFBP2, MAGE.A4, BMI1, GAD2, FXR1, HuC, CRYAA, ESO1) in 332 sera from patients with early-stage NSCLC and 375 control samples in the training set samples. The meaning of "***" indicates that the two sets of data have a very significant difference (P < 0.001).

T-test was conducted for the levels of 36 autoantibodies in 332 sera from patients with early-stage NSCLC and 375 control samples (including 167 sera from patients with benign lung lesions and 208 sera from subjects with no obvious lung abnormalities found in physical examination) in the training set using 36 lung cancer antigens *via* Luminex liquid chip technique. Finally, 12 autoantibodies corresponding to the antigens were obtained, and their levels in lung cancer sera were significantly higher than those in normal human sera, indicating that these antigens have certain diagnostic capabilities for lung cancer. Regarding the lung cancer antigens that had a significant diagnostic capability for lung cancer samples: ZNF573, BRAF, TM4SF1, SOX2, IGFBP2, MAGE.A4, BMI1, GAD2, FXR1, HuC, CRYAA, ESO1, the distribution levels of those tested autoantibodies in the lung cancer group and the control group are shown in **Figure 4**.

#### 3.4.2 Selection of optimal lung cancer antigen combination

**Figure 5:**
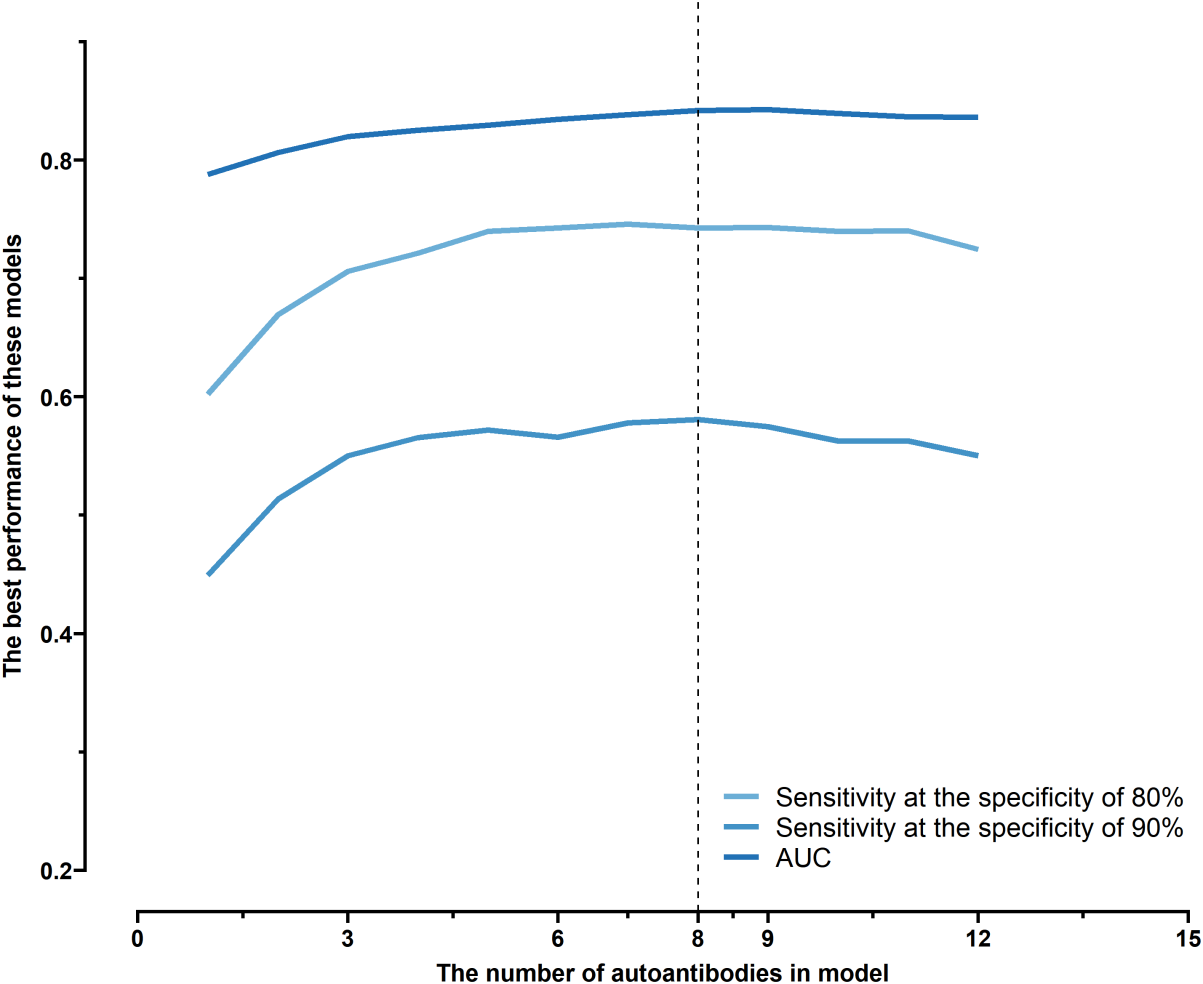
Optimal sensitivity at 80/90% specificity and AUC achieved by logistic models composed of different numbers of autoantibodies under 5-fold cross-validation.

**Figure 6:**
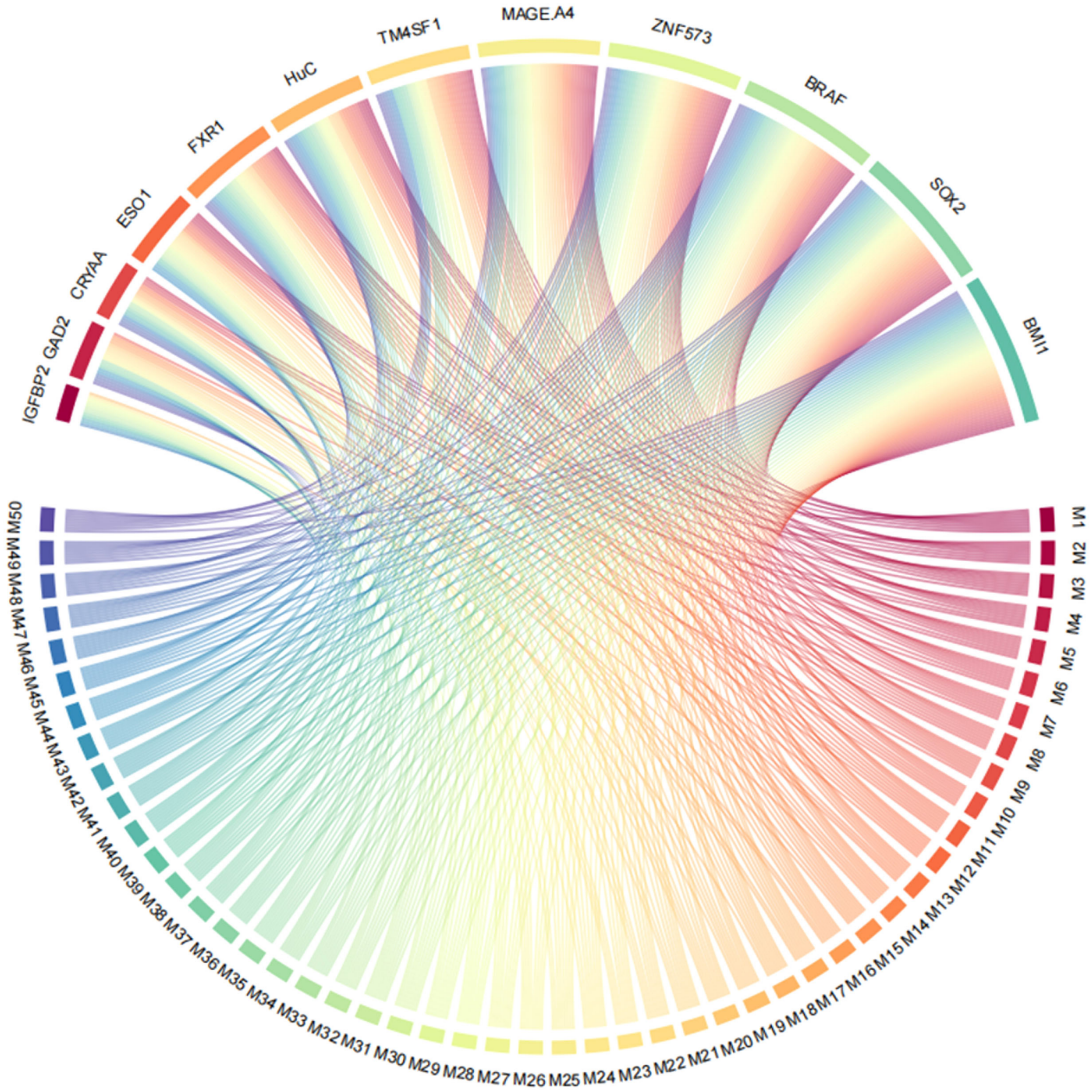
Use of ZNF573, BRAF, TM4SF1, SOX2, IGFBP2, MAGE.A4, BMI1, GAD2, FXR1, HuC, CRYAA, and ESO1 in top 50 models in terms of AUC based on cross-validation.

**Figure 7:**
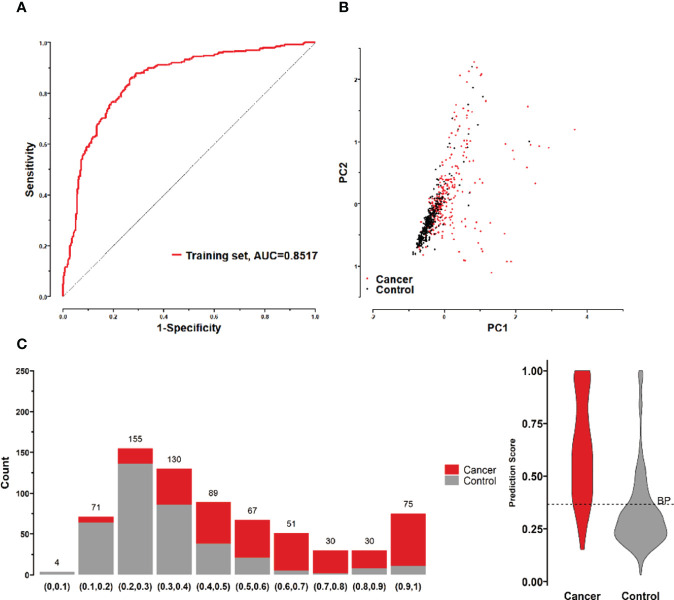
**(A)** ROC curves of 8 lung cancer antigens (ZNF573, BRAF, TM4SF1, SOX2, MAGE.A4, BMI1, FXR1, and HuC) in the training set; **(B)** Principal component analysis of 8 lung cancer antigens (ZNF573, BRAF, TM4SF1, SOX2, MAGE.A4, BMI1, FXR1, and HuC) in the training set samples, and scatter plots using principal component 1 and principal component 2 as coordinates for samples in control and tumor groups. **(C)** Distribution of the prediction scores for the logistic regression model fitted to 8 lung cancer antigens in the training set.

All possible combinations(consisting of 1, 2, 3, and so on, until 12 autoantibodies)of lung cancer autoantibodies were exhausted, and different logistic models were built individually according to those combinations. Based on 5-fold cross-validation, the AUC and the sensitivity index at 90% and 80% specificity of logistic model were calculated. It was found that, under 5-fold cross-validation, the maximum AUC and sensitivity at 90% and 80% specificity of the logistic model did not increase anymore with the number of lung cancer autoantibodies in the model once the number reached 8. The results are shown in **Figure 5**. Thus, the optimal number of lung cancer autoantibodies in the model was 8. Further analysis revealed that, for the exhaustive list of all possible models consisting of 8 lung cancer autoantibodies among the 12 markers, the 8 lung cancer autoantibodies, targeting ZNF573, BRAF, TM4SF1, SOX2, MAGE.A4, BMI1, FXR1, and HuC respectively, were most frequently used out of the top 50 models in terms of AUC based on 5-fold cross-validation. The results are shown in **Figure 6**. It indicates that these 8 lung cancer autoantibodies are of great importance to the model. **Table 6** shows the top 10 models in terms of AUC consisting of 8 lung cancer autoantibodies based on 5-fold cross-validation. It can be concluded from the table that model 1 (ZNF573, BRAF, TM4SF1, SOX2, MAGE.A4, BMI1, FXR1, HuC) has the greatest AUC, suggesting that the combination of the 8 lung cancer autoantibodies in model 1 is the optimal model. The ROC curve of model 1 is shown in **Figure 7A**, which has an AUC of 0.8517, a sensitivity of 76.75% at 80% specificity, and a sensitivity of 59.02% at 90% specificity. Subsequently, the principal components analysis was conducted for the samples in the training set using the abundance of these 8 lung cancer autoantibodies. As shown in **Figure 7B**, the tumor samples and control samples in the training set have remarkably different distributions in the scatter plot with principal component 1 and principal component 2 as the coordinates, indicating that these 8 lung cancer autoantibodies can effectively distinguish tumor samples from control samples. In terms of the prediction score(the possibility of being sera from patients with early-stage NSCLC predicted by the model fitted by these 8 lung cancer autoantibodies), the prediction scores were dominated by the control group samples in the interval of (0.00–0.4) and by the tumor group samples in the interval of (0.40–1.00). The results are shown in **Figure 7C**.

**Table 6:** Top 10 logistic models consisting of 8 lung cancer antigen markers in terms of AUC and their sensitivity at 80/90% specificity and AUC under 5-fold cross-validation.

Serial No.	Marker combination	Sensibility at 80% specificity	Sensibility at 90% specificity	AUC
Model 1	ZNF573 + BRAF + TM4SF1 + SOX2 + MAGE.A4 + BMI1 + FXR1 + HuC	0.7311	0.5507	0.8420
Model 2	BRAF + TM4SF1 + SOX2 + MAGE.A4 + BMI1 + FXR1 + HuC + ESO1	0.7251	0.5418	0.8400
Model 3	ZNF573 + BRAF + TM4SF1 + SOX2 + MAGE.A4 + BMI1 + FXR1 + ESO1	0.7217	0.5382	0.8398
Model 4	ZNF573 + BRAF + SOX2 + MAGE.A4 + BMI1 + FXR1 + HuC + ESO1	0.7187	0.5599	0.8394
Model 5	ZNF573 + BRAF + TM4SF1 + SOX2 + MAGE.A4 + BMI1 + HuC + ESO1	0.7340	0.5810	0.8392
Model 6	ZNF573 + BRAF + TM4SF1 + SOX2 + MAGE.A4 + BMI1 + FXR1 + CRYAA	0.7034	0.5444	0.8368
Model 7	ZNF573 + BRAF + TM4SF1 + SOX2 + BMI1 + FXR1 + HuC + ESO1	0.7341	0.5476	0.8366
Model 8	ZNF573 + BRAF + TM4SF1 + SOX2 + MAGE.A4 + BMI1 + HuC + CRYAA	0.7249	0.5598	0.8354
Model 9	ZNF573 + BRAF + TM4SF1 + SOX2 + MAGE.A4 + BMI1 + GAD2 + FXR1	0.7217	0.5139	0.8354
Model 10	ZNF573 + BRAF + TM4SF1 + MAGE.A4 + BMI1 + FXR1 + HuC + ESO1	0.7340	0.5416	0.8352

#### 3.4.3 Validation of optimal lung cancer antigen combination

**Figure 8:**
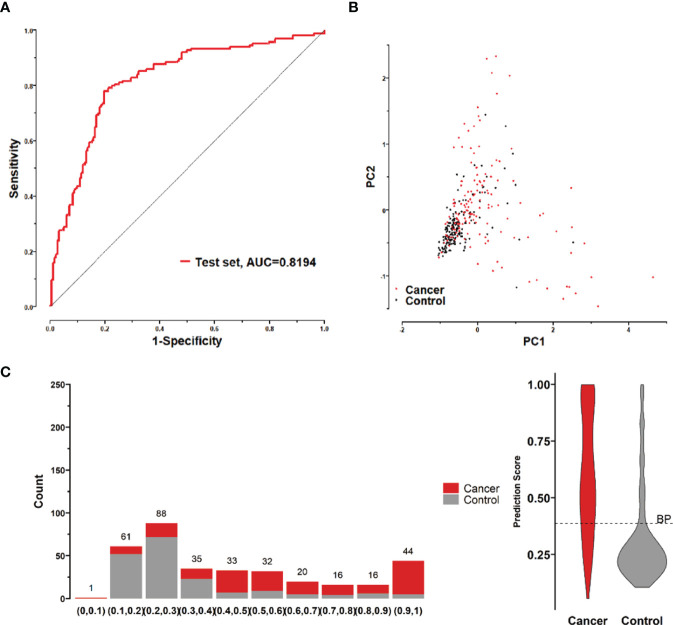
**(A)** ROC curve of the logistic model composed of 8 lung cancer antigens in the test set; **(B)** Principal component analysis of the test set samples in terms of the abundance of 8 lung cancer antigens, and scatter plots with the principal component 1 and principal component 2 as coordinates for samples in the control and tumor groups. **(C)** Distribution of the prediction scores in the test set for the logistic model composed of 8 lung cancer antigens.

**Figure 9:**
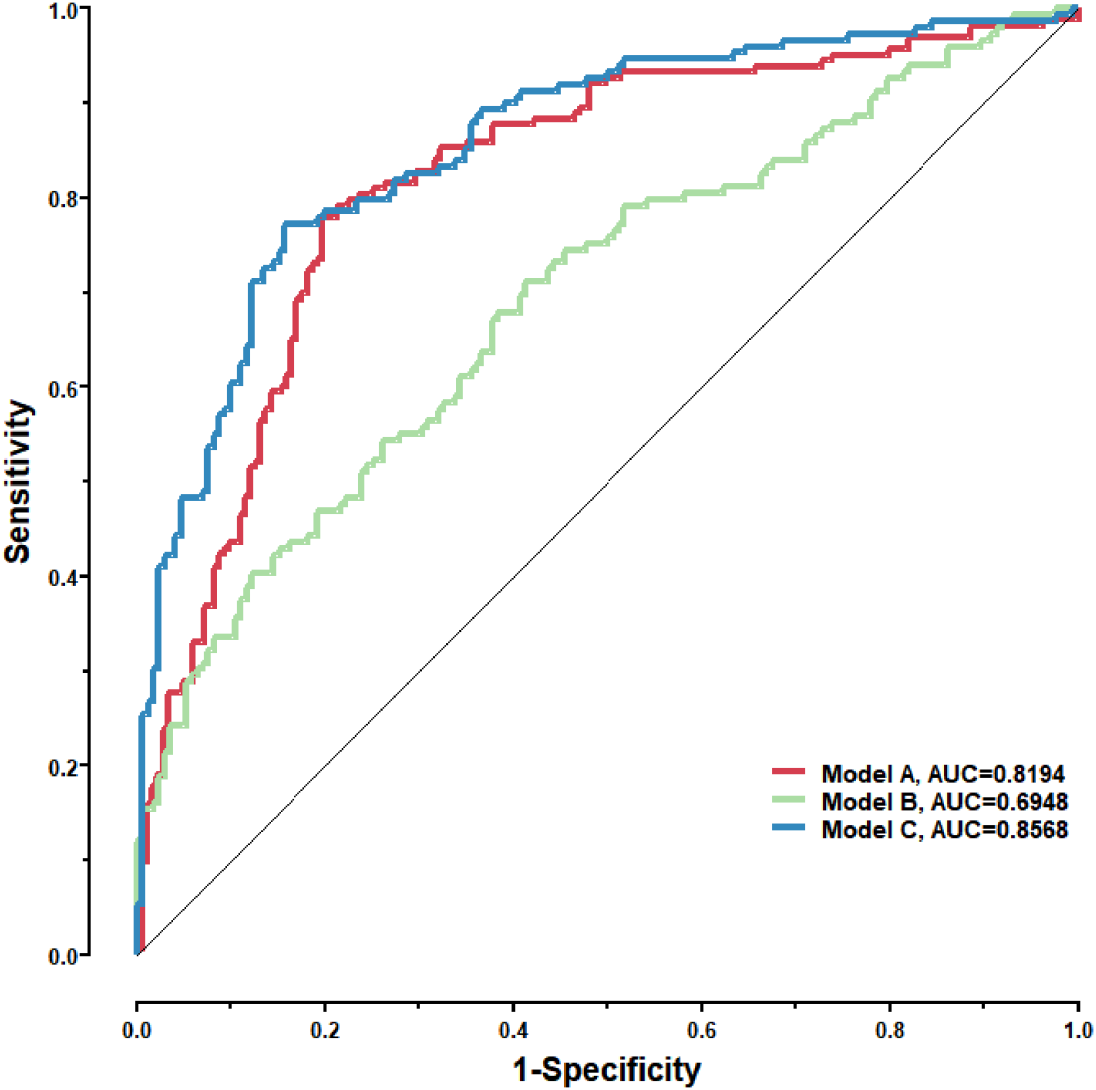
Model A: The logistic model consisting of the 8 lung cancer antigens; Model B: The logistic model consisting of 4 conventional serum tumor markers; Model C: The logistic model consisting of the 8 lung cancer antigens and the 4 conventional serum tumor markers.

To further validate the diagnostic efficacy of the model consisting of the 8 lung cancer autoantibodies identified in this study in distinguishing early-stage NSCLC, additional serum samples were collected from 163 patients with early-stage NSCLC and 183 patients with benign lung lesions and used as external test set samples to examine the diagnostic capability of the model in the test set samples. The results showed that the model in the test set samples had a ROC curve with AUC = 0.8194 and a sensitivity of 43.56% at 90% specificity and 77.91% at 80% specificity (**Figure 8A**). A principal component analysis was conducted for the abundance of the 8 lung cancer autoantibodies in the test set (**Figure 8B**). As shown in the figure, there were relatively remarkable differences in the distribution of tumor samples and control samples in the test set in the scatter plot with principal component 1 and principal component 2 as coordinates, indicating that the 8 lung cancer autoantibodies could effectively distinguish tumor samples from control samples in the test set. Regarding the prediction score of the model fitted by the 8 lung cancer autoantibodies in the test set (**Figure 8C**), the prediction scores were dominated by the control group samples in the interval (0.00–0.4) and by the tumor group samples in the interval (0.40–1.00). In summary, the model fitted to the 8 lung cancer autoantibodies in the training set also performed well in the test set, which could effectively distinguish tumor samples from control samples in the test set. For the test set samples, additional data were obtained for 4 conventional serum tumor markers, CYFRA21-1, CEA, SCCA, and NSE, and a binary logistic regression model was developed for these 4 markers, which had an AUC of 0.6948, a sensitivity of 33.56% at 90% specificity, and a sensitivity of 46.98% at 80% specificity (**Figure 9B**). The combination of these 4 conventional serum tumor markers was remarkably less efficient than the 8 lung cancer autoantibodies for the diagnosis of lung cancer (**Figure 9A**). Subsequently, we built another logistic model whose independent variables consist of both the 8 lung cancer autoantibodies and the 4 serum tumor markers. the diagnostic efficiency of the new model for lung cancer exhibited further improvement, with an AUC of 0.8568 and a sensitivity of 60.4% at 90% specificity (**Figure 9C**). Therefore, the 4 conventional serum tumor markers, CYFRA21-1, CEA, SCCA, and NSE, were suggested to be effective supplements for the 8 lung cancer autoantibodies obtained in this study in distinguishing early-stage NSCLC.

## 4 Discussion

A number of experimental results in recent years have shown that tumor antigens can not only mediate the production of cellular immunity but also induce the production of specific serum antibodies in tumor patients (20). The SEREX technique established by Sahin et al. is a powerful tool for the identification of tumor antigens, which isolates tumor antigens by screening the cDNA expression library of tumor tissues with the help of autoantibodies in patient serum. Tumor antibodies in the body are more stable and easier to detect than tumor antigens, so the search for tumor antigens by SEREX technology and the use of corresponding tumor antigen-associated antibodies as markers have become one of the approaches to serological diagnosis of tumors (18, 21). Therefore, this study was designed to search for tumor antigens of early-stage lung cancer *via* SEREX and to perform early screening and diagnosis of lung cancer by detecting their corresponding antibodies. However, considering the complex pathological types of lung cancer along with the heterogeneity of tumors, the antigens obtained by screening from single tissue or single autologous serum may be rather inadequate for tumor diagnosis. To overcome these difficulties, the mRNA abundance of single tissue was diluted using mixed tissues from 10 early-stage lung cancer tissues of different pathological types, which made it more convenient to screen antigens commonly expressed in lung cancer tissues from different individuals and in different pathological types. At the same time, multiple autologous and mixed allogeneic sera from different pathological types of lung cancer were used for library screening, which reduced the titers of autoantibodies in individual serums and thus increased the relative titers of common antibodies in different individual serums. As a result, the possible heterogeneity of early-stage lung cancer was overcome in these ways. In addition, there are plenty of naturally occurring antibodies inherent in the human body (22), and antibodies produced by unnoticed mild infections or antigenic stimuli are widely prevalent in daily life. For instance, various types of heterophilic antibodies have been demonstrated in numerous studies to bind to animal immunoglobulins used in immunoassays, thus interfering with serum immunoassays (23, 24). Furthermore, most natural antibodies can react with one or more autoantigens (25), and by interacting with various self-components present in the organism, natural antibodies establish an extensive dynamic network that contributes to the general homeostasis of the organism (26). Therefore, positive clones identified by SEREX screening may include those that react with natural antibodies. To reduce false positive clones being screened, a second-round screening was conducted for all positive clones, and of the 54 clones initially identified as positive for reactions with cancer sera, 18 showed higher reactions with healthy sera. These clones were excluded due to the possibility that they might react with some common natural antibodies. The second-round screening allows these pre-screened tumor-associated autoantibodies with higher diagnostic specificity.

The sequences in phage display system may be relatively short peptides rather than full-length proteins, as seen in other literature (27). In addition, since the phage display system is based on a prokaryotic expression platform, the expressed tumor antigens are deficient in modification processes (such as glycosylation) commonly found in higher organisms. The absence of glycosylation may even alter the immunogenicity of the antigens (28–30), and therefore, the antibodies screened may have low specificity or low affinity. Some studies showed that, with eukaryotic display systems such as yeast or mammalian cells (31, 32), it is possible to screen for high-affinity antibodies with well-defined biological specificities. And this is also one of our improvement goals afterward.

TAAs have been studied in the past for the early diagnosis of lung cancer, but ELISA assays were applied in these studies; this method made it difficult to increase the number of indicators and had low throughput, narrow linearity range, and poor precision. Therefore, the screening and matching of markers from a larger panel to improve the sensitivity and specificity of diagnosis is one of the directions in the field of early diagnosis of lung cancer. In this study, a liquid protein chip for simultaneous detection of antibodies to 36 autoantigens was constructed and used to test a training set consisting of 707 samples. 12 significant markers were screened and finally optimized to obtain the marker combination made up of 8 lung cancer autoantibodies, including those targeting ZNF573, BRAF, SOX2, MAGE.A4, BMI1, FXR1, HuC, and ESO1. This combination had the highest diagnostic performance. The obtained marker combination and its model were validated in an independent test set consisting of 346 samples. By combining these 8 lung cancer autoantibodies with the 4 tumor markers (CYFRA21-1, CEA, SCCA, and NSE) in the test set, the sera from patients with early-stage lung cancer and the sera from patients with benign lung nodules were better differentiated. Therefore, the combination of early lung cancer autoantibodies and several serum tumor markers commonly used in clinical practice, namely CYFRA21-1, CEA, SCCA, and NSE, can further enable effective differentiation between patients with early lung cancer and patients with benign lung nodules. Model C in this study, compared with Oncimmune’s EarlyCDT, showed further improvement in sensitivity and specificity. Thus, the combination of 8 lung cancer autoantibodies obtained in this study and 4 serological markers is advantageous in the early diagnosis of lung cancer.

In conclusion, a set of lung cancer autoantibodies with high diagnosis efficiency for early-stage NSCLC were identified through SEREX method and screened based on liquid chip technique. The detection rate of lung cancer was further increased by combining these lung cancer antigens with the 4 conventional serum tumor markers. This study provides valuable reference of markers for the detection of early-stage lung cancer.

## Data availability statement

The raw data supporting the conclusions of this article will be made available by the authors, without undue reservation.

## Ethics statement

The studies involving human participants were reviewed and approved by Medical Ethics Committee, Tongji Medical College, Huazhong University of Science and Technology. The patients/participants provided their written informed consent to participate in this study.

## Author contributions

Conceptualization: CH, NZ, RC, FZ. Methodology: CH, NZ, RC, FZ, HZ. Investigation: CH, NZ, RC, FZ, HZ, ZW, WX, DL, JC, LZ, LH. Data Analysis: CH, FZ, ZW. Resources: CH, NZ, RC, FZ, DL. Writing: CH, NZ, RC. All authors contributed to the article and approved the submitted version.

## Acknowledgments

Special thanks to the Talent Work Leading Group of Guangzhou Development Zone, Huangpu District, Guangzhou City, Guangdong Province, China. This research was funded by the Project of Leading Talents for Entrepreneurship of Guangzhou Development Zone, Huangpu District, Guangzhou City (Document No.3 of Suipoa Talent Line [2019]).

## Conflict of interests

Author CH, FZ, ZW, DL, LH, WX, JC and LZ were employed by Guangzhou BioBlue Technology Co. Ltd.

The remaining authors declare that the research was conducted in the absence of any commercial or financial relationships that could be construed as a potential conflict of interest.

## Publisher’s note

All claims expressed in this article are solely those of the authors and do not necessarily represent those of their affiliated organizations, or those of the publisher, the editors and the reviewers. Any product that may be evaluated in this article, or claim that may be made by its manufacturer, is not guaranteed or endorsed by the publisher.
