# Supplementary material for: A tumor-associated autoantibody panel for the detection of non-small cell lung cancer
Source: Front Oncol. 2022 Dec 2;12:1056572. doi: 10.3389/fonc.2022.1056572 (PMC9757608; doi:10.3389/fonc.2022.1056572)
Supplement: Supplementary file 1 [file DataSheet_1.docx]

**Table S1. Functional roles of 12 Tumor associated antigens.**

| NO. | Name | Full name | Summary |
| --- | --- | --- | --- |
| 1 | ZNF573 | Zinc finger 573 | A zinc-finger protein consisting of 645 amino acids, it is a zinc-finger protein that inhibits the activation of nuclear transcription factor-kappa B (NF-kappa B) and c-Jun amino-terminal kinase (c-Jun Nterminalkinase) induced by TRAF6. It plays an important role in osteoclast differentiation by regulating the signaling activity of TRAF6 (1). |
| 2 | BRAF | V-raf murine sarcoma viral oncogene homolog B1 | BRAF, a member of the RAF gene family, its translated BRAF protein consists of 783 amino acids and possesses three conserved protein structural domains. the BRAF gene is a downstream signaling molecule belonging to the RAS and encodes a kinase that is a key component of the RAS/RAF/MEK/MARK signaling pathway. When a mutation occurs, the BRAF mutant protein will exhibit a state of sustained activation, resulting in unrestricted cell division and proliferation, leading to tumorigenesis and development (2, 3). |
| 3 | TM4SF1 | Transmembrane-4 -L-six-family member 1 | TM4SF1, also known as TAAL6 and L6, belongs to the TM4SF family. The molecular structure of the TM4SF family is characterized by four highly conserved hydrophobic transmembrane regions.TM4SF1 is mainly involved in tumor cell growth, adhesion, invasion, and metastasis, and is positively correlated with cell invasive ability and poor prognosis.TM4SF1 is highly expressed in human lung, colon, and breast cancer cells. High expression of the TM4SF1 gene in lung cancer patient specimens is significantly associated with early postoperative recurrence and short survival (4, 5). |
| 4 | SOX2 | Sex determining region Y-box2 | SOX2 is a stem cell transcription factor with an extremely important role in the development of squamous lung carcinoma, associated with squamous epithelial differentiation of squamous carcinoma, induces the expression of squamous carcinoma markers p63 and keratin 6, activates downstream epidermal growth factor receptors and insulin-like growth factor-1, thereby promoting proliferation, invasion and migration of cancer cells. SOX2 plays an important role in the infiltration and metastasis of NSCLC, and tumors with high SOX2 expression are more malignant and are independent predictors of poor prognosis (6, 7). |
| 5 | IGFBP2 | Insulin like growth factor binding protein 2 | The protein encoded by this gene is one of six similar proteins that bind insulin-like growth factors I and II (IGF-I and IGF-II). High expression levels of this protein promote the growth of several types of tumors and may be predictive of the chances of recovery of the patient. Several transcript variants, one encoding a secreted isoform and the others encoding nonsecreted isoforms, have been found for this gene.  IGFBP2 expression was associated with worse prognosis in several tumors, and may serve as a potential prognostic biomarker in cancer patients (8). |
| 6 | MAGE A4 | Melanoma antigen family A4 | MAGE A4 belongs to the MAGE family, a tumor-specific testicular cancer antigen, which is highly expressed in malignant tumors, such as lung cancer, and low or no expression in normal tissues. MAGE A4 is involved in apoptosis and cell cycle processes. Its high expression also affects the sensitivity of chemotherapeutic agents to apoptosis in malignant tumors (9, 10). |
| 7 | BMI1 | B cell-specific Moloney murine leukemia virus integration site 1 | BMI1 is a major member of the PCG family. As a proto-oncogene, BMI1 is located at the source of regulation of cell differentiation and is highly expressed in a variety of malignancies, including stem cells and lung and brain, and plays a key role in tumor cell proliferation, senescence, cell cycle and apoptosis, regulation of chromosomal stability and self-renewal ability.BMI1 is overexpressed in a variety of malignancies of epithelial cell origin, including non-small cell lung cancer, and its expression level is strongly correlated with tumor malignancy, invasion and metastasis and prognosis (11). |
| 8 | GAD2 | Glutamate decarboxylase 2 | GAD2 protein is one of several forms of glutamate decarboxylase, approximately 65 K da, primarily responsible for catalyzing the production of -aminobutyric acid from l -glutamate, an autoantigen in insulin-dependent diabetes mellitus that is highly expressed in the brain and pancreas (12). |
| 9 | FXR1 | Fragile X autosomal homolog 1 | FXR1 is a member of the Fragile X mental retardation-associated gene family, which encodes a protein that binds RNA, shuttles between the nucleus and cytoplasm, and is associated with polyribosomes. FXR1 has two heterogeneous nuclear ribonucleoprotein K regions (KH) and an RGG box that binds to artificial RNA homodimers in vitro. FXR1 functions as a cell growth-dependent translational activity, the FXR1 gene has a high rate of overexpression in squamous cell carcinomas, and FXR1 regulation of translational activity is associated with cell carcinogenesis (13, 14). |
| 10 | HuC | ELAV like RNA binding protein 3 | Also known as ELAVL3, a member of the ELAVL protein family, is a protein essential for the extension of growth cone protrusion formation and survival of mature neurons during neuronal embryonic development, and is specifically expressed throughout the development of peripheral and central neurons(15). Additional studies have shown that some neuroendocrine-related tumors such as small cell lung cancer and neuroblastoma are also expressed (16). |
| 11 | CRYAA | alpha A crystallin | Mammalian lens crystallins are divided into the α, β and γ families. α crystallins are induced by heat shock and are members of the small heat shock protein (HSP20) family. these non-homogeneous aggregates consist of 30-40 subunits The other two functions of α crystallins are auto kinase activity and involvement in intracellular structures. this encoded protein has been identified as a part-time protein based on its ability to perform different mechanistic functions. defects in this gene result in autosomal dominant hereditary cataracts (ADCC) (17). |
| 12 | ESO1 | Esophageal squamous cell carcinoma 1 | ESO1, also known as NY-ESO-1, is a tumor-specific antigen belonging to the cancer testis antigen (CTA) family, which can stimulate both cellular and humoral immunity in tumor patients and is considered to be one of the most immunogenic tumor/testis antigens NY-ESO-1 expression is associated with tumor tissue grading, tumor development and prognosis, and is of great value in the early diagnosis of tumors, monitoring of tumor development, and evaluation of treatment effects (18, 19). |

**Table S2. GenBank accession numbers of 12 Tumor associated antigens.**

| NO. | Name | Full name | GenBank accession number |
| --- | --- | --- | --- |
| 1 | ZNF573 | Zinc finger 573 | OP680444 |
| 2 | BRAF | V-raf murine sarcoma viral oncogene homolog B1 | OP680445 |
| 3 | TM4SF1 | Transmembrane-4 -L-six-family member 1 | OP680446 |
| 4 | SOX2 | Sex determining region Y-box2 | OP680447 |
| 5 | IGFBP2 | Insulin like growth factor binding protein 2 | OP680448 |
| 6 | MAGE A4 | Melanoma antigen family A4 | OP680449 |
| 7 | BMI1 | B cell-specific Moloney murine leukemia virus integration site 1 | OP680450 |
| 8 | GAD2 | Glutamate decarboxylase 2 | OP680451 |
| 9 | FXR1 | Fragile X autosomal homolog 1 | OP680452 |
| 10 | HuC | ELAV like RNA binding protein 3 | OP680453 |
| 11 | CRYAA | alpha A crystallin | OP680454 |
| 12 | ESO1 | Esophageal squamous cell carcinoma 1 | OP680455 |

**Table S3. Sample detection data location of the training and test sets.**

| NO. | Database | Digital Object Identifier (DOI) |
| --- | --- | --- |
| 1 | Figshare (https://figshare.com) | 10.6084/m9.figshare.21308697 |

1. Shin JN, Kim I, Lee JS, Koh GY, Lee ZH, Kim HH. A novel zinc finger protein that inhibits osteoclastogenesis and the function of tumor necrosis factor receptor-associated factor 6. The Journal of biological chemistry. 2002;277(10):8346-53.

2. Ji H, Wang Z, Perera SA, Li D, Liang MC, Zaghlul S, et al. Mutations in BRAF and KRAS converge on activation of the mitogen-activated protein kinase pathway in lung cancer mouse models. Cancer Res. 2007;67(10):4933-9.

3. Dankort D, Filenova E, Collado M, Serrano M, Jones K, McMahon M. A new mouse model to explore the initiation, progression, and therapy of BRAFV600E-induced lung tumors. Genes Dev. 2007;21(4):379-84.

4. Allioli N, Vincent S, Vlaeminck-Guillem V, Decaussin-Petrucci M, Ragage F, Ruffion A, et al. TM4SF1, a novel primary androgen receptor target gene over-expressed in human prostate cancer and involved in cell migration. Prostate. 2011;71(11):1239-50.

5. Shih SC, Zukauskas A, Li D, Liu G, Ang LH, Nagy JA, et al. The L6 protein TM4SF1 is critical for endothelial cell function and tumor angiogenesis. Cancer Res. 2009;69(8):3272-7.

6. Cancer Genome Atlas Research N. Comprehensive genomic characterization of squamous cell lung cancers. Nature. 2012;489(7417):519-25.

7. Yoon HI, Park KH, Lee EJ, Keum KC, Lee CG, Kim CH, et al. Overexpression of SOX2 Is Associated with Better Overall Survival in Squamous Cell Lung Cancer Patients Treated with Adjuvant Radiotherapy. Cancer research and treatment. 2016;48(2):473-82.

8. Zhang B, Hong CQ, Luo YH, Wei LF, Luo Y, Peng YH, et al. Prognostic value of IGFBP2 in various cancers: a systematic review and meta-analysis. Cancer medicine. 2022;11(16):3035-47.

9. Fujiwara-Kuroda A, Kato T, Abiko T, Tsuchikawa T, Kyogoku N, Ichinokawa M, et al. Prognostic value of MAGEA4 in primary lung cancer depends on subcellular localization and p53 status. Int J Oncol. 2018;53(2):713-24.

10. Peikert T, Specks U, Farver C, Erzurum SC, Comhair SA. Melanoma antigen A4 is expressed in non-small cell lung cancers and promotes apoptosis. Cancer Res. 2006;66(9):4693-700.

11. Vrzalikova K, Skarda J, Ehrmann J, Murray PG, Fridman E, Kopolovic J, et al. Prognostic value of Bmi-1 oncoprotein expression in NSCLC patients: a tissue microarray study. J Cancer Res Clin Oncol. 2008;134(9):1037-42.

12. Bedi S, Richardson TM, Jia B, Saab H, Brinkman FSL, Westley M. Similarities between bacterial GAD and human GAD65: Implications in gut mediated autoimmune type 1 diabetes. PloS one. 2022;17(2):e0261103.

13. Yang CX, Wright EC, Ross JW. Expression of RNA-binding proteins DND1 and FXR1 in the porcine ovary, and during oocyte maturation and early embryo development. Mol Reprod Dev. 2012;79(8):541-52.

14. Comtesse N, Keller A, Diesinger I, Bauer C, Kayser K, Huwer H, et al. Frequent overexpression of the genes FXR1, CLAPM1 and EIF4G located on amplicon 3q26-27 in squamous cell carcinoma of the lung. Int J Cancer. 2007;120(12):2538-44.

15. Ogawa Y, Kakumoto K, Yoshida T, Kuwako KI, Miyazaki T, Yamaguchi J, et al. Elavl3 is essential for the maintenance of Purkinje neuron axons. Scientific reports. 2018;8(1):2722.

16. Hatanaka T, Higashino F, Tei K, Yasuda M. The neural ELAVL protein HuB enhances endogenous proto-oncogene activation. Biochem Biophys Res Commun. 2019;517(2):330-7.

17. Schimansky A, Yadav JK. Amyloid cross-sequence interaction between Aβ(1-40) and αA(66-80) in relation to the pathogenesis of cataract. International journal of biological macromolecules. 2021;179:61-70.

18. Konishi J, Toyooka S, Aoe M, Omura Y, Washio K, Tsukuda K, et al. The relationship between NY-ESO-1 mRNA expression and clinicopathological features in non-small cell lung cancer. Oncology reports. 2004;11(5):1063-7.

19. Yang J, Jiao S, Kang J, Li R, Zhang G. Application of serum NY-ESO-1 antibody assay for early SCLC diagnosis. Int J Clin Exp Pathol. 2015;8(11):14959-64.
